# Healthcare providers' intention to discriminate against people with HIV

**DOI:** 10.3389/fpubh.2025.1464250

**Published:** 2025-03-14

**Authors:** Almutaz M. Idris, Rik Crutzen, Hubertus W. van den Borne, Sarah E. Stutterheim

**Affiliations:** ^1^College of Applied Medical Science, Buraydah Colleges, Buraydah, Saudi Arabia; ^2^Department of Health Promotion, Care and Public Health Research Institute, Maastricht University, Maastricht, Netherlands

**Keywords:** stigma, healthcare providers, people with HIV, patient with HIV, discrimination, Sudan

## Abstract

**Background:**

Healthcare providers' discrimination practices against people with HIV is a real challenge for control and prevention efforts. The study aims to explore the association between healthcare providers' intention to discriminate against people with HIV and HIV stigma-related constructs, their sociodemographic, and occupation characteristics in Sudan.

**Methods:**

A cross-sectional survey of healthcare providers was carried out in governmental hospitals in Kassala State, Sudan. Respondents completed measures assessing their intentions to discriminate against people with HIV, HIV-related stigma constructs, sociodemographic, and occupational characteristics. Bivariate and multiple linear regression analysis were used to assess the associations between discriminatory intentions against people with HIV and the studied variables.

**Results:**

A total of 387 participants (223 physicians and 164 nurses) completed the survey. Participants had relatively high intentions to discriminate against people with HIV (M = 5.19, SD = 1.34—on a scale from 1 to 7), prejudiced attitudes (M = 4.70, SD = 1.29), internalized shame about HIV (M = 5.19, SD = 1.34), fear of HIV (M = 4.65, SD = 1.39), and the belief that patients with HIV do not deserve good care (M = 4.90, SD = 1.35). Healthcare providers' intention to discriminate against people with HIV was associated with prejudiced attitudes, internalized shame about HIV, fear of HIV, and the belief that people with HIV do not deserve good care. Female health care providers, nurses, and those with postgraduate degrees and fewer years of work experience were more likely to have a high intention to discriminate against people with HIV.

**Conclusions:**

Intention to discriminate against people with HIV was high among healthcare providers. Addressing HIV-related stigma constructs and understanding the differential effects of healthcare providers' sociodemographic and occupational characteristics on their discriminatory intentions are imperative to developing effective intervention to reduce intention to discriminate against people with HIV among healthcare providers.

## Background

HIV stigma is a well-documented barrier for controlling HIV epidemic worldwide (1, 2). It also poses significant challenge to the achievement of global HIV-related treatment and prevention targets (3–5). In Sudan like other countries HIV stigma and discrimination is against people with HIV is high (6, 7) and only 37% of the estimated people with HIV knew their status, and about 22% were on treatment in 2019 (8).

HIV stigma is a process by which people with HIV are socially disregarded, discredited, and discriminated against because of their HIV condition or identity (9–11). This process comprises various interconnected components (12), such as prejudiced attitudes toward people with HIV, negative feelings of associating with or caring for people with HIV, and fearing people with HIV or HIV infection (13–15), discrimination which is final result of stigmatization (16). People with HIV could internalize or anticipate stigmatization and discrimination practices against them (17, 18). Internalized stigma refers to people with HIV accepting negative beliefs and notions from others and applying them to themselves (19). Anticipated stigma refers to people with HIV believing that others will treat them negatively because of their HIV status (20). Moreover, HIV stigma can occur in different settings, including the community and healthcare (21, 22).

In health care settings, healthcare providers stigmatization and discriminatory behaviors can take different forms including testing HIV without consent (17), disclosing HIV results to others (23), blaming for getting HIV infection, providing different treatment, refusing to treat, or unnecessarily referring to other health care providers, and using extra precautions when managing people with HIV (24–26).

Healthcare providers discrimination toward people with HIV can be particularly damaging as it directly influences HIV treatment and prevention and people with HIV health outcomes (23, 27). For example, discrimination of people with HIV by healthcare providers may result in failure to provide HIV services, delay engagement in care, poor adherence to treatment and refusal of services (28–35). Among people with HIV discrimination related practices are associated with poor mental health outcomes (36–39).

People's intentions are a proxy for their behavior (40, 41). The more a person has the intention to engage in a specific behavior, such as discrimination against people with HIV, the more likely it is to perform the behavior. Many factors can drive healthcare providers' discriminatory intentions against people with HIV. For example, Steward and colleagues (42) found that negative moral judgments toward people with HIV were associated with a higher intention to discriminate against people with HIV among healthcare providers in India. Another study among Indian nurses showed that fear of HIV transmission was associated with intentions to discriminate against people with HIV (43).

HIV stigma-related constructs can also influence healthcare providers' intentions to discriminate against people with HIV. Previous study of the physicians' intention to discriminate against people with HIV in Malaysia (44), found that fear of HIV, prejudiced attitudes toward HIV, HIV-related shame, and beliefs that patients with HIV do not deserve good care were associated with discriminatory intentions among healthcare providers.

Healthcare providers' sociodemographic characteristics can also influence their intentions to discriminate against people with HIV. For example, female medical students in Malaysia were more likely than male students to discriminate against people with HIV (45). Studies have also identified occupational characteristics of healthcare providers, such as physician, nurse, and years of work experience, as drivers behind healthcare providers' intentions to discriminate against people with HIV (46, 47).

However, in Sudan, there is a lack of knowledge healthcare providers' intentions to discriminate against people with HIV and its associated factors. In addition, existing information on stigma and discrimination among healthcare providers is limited. This study aims to explore what are the sociodemographic and occupational characteristics, and stigma-related constructs associated with healthcare providers' intention to discriminate people with HIV in Kassala State, Sudan. Such information will be useful and would provide insights into discrimination reduction interventions targeting healthcare providers.

## Methods

The STROBE Statement of cross-sectional studies (48) guides the description of the methods in this study.

### Study design and settings

The study design was a cross-sectional survey of healthcare providers in governmental hospitals in Kassala state. The state is in the eastern of Sudan and is divided into 11 localities. The state has five referral governmental hospitals, 14 rural hospitals, and several private hospitals and health centers spread throughout its localities. These health facilities provide healthcare services to about 2.9 million people (i.e., the state' estimated population). Estimated HIV prevalence in Kassala state was 4.18% in 2018, which is higher than the prevalence in other states in Sudan (49). The state shares borders with two African countries that have high HIV prevalence (50). Furthermore, the state is characterized by a high HIV prevalence among female sex workers and tuberculosis patients (51, 52). According to Kassala Ministry of Health reports, the provision of HIV-related services started in 2009 in selected health facilities.

The study's ethical clearance was obtained from the Kassala State Ministry of Health Research Ethics Committee. Permits were granted by administrative authorities in the hospitals included in the study.

### Participants and procedures

The study sample size was computed using the single proportion formula for sample size estimation, with a prevalence of 0.5 and a margin of error of 0.05. This resulted in a minimal required sample of 384 from the study population. However, the study included 387 participants. We used a multi-stage sampling technique to select hospitals and participants. Simple random sampling was used to select 11 rural hospitals and three referral hospitals from the state. Then, in each selected hospital, we used a systematic sampling method to randomly select participants from the available hospital staff list. The first participant was chosen randomly, and then an interval was employed to select the others. The interval was determined based on the healthcare provider lists and the desired sample size per hospital. We replaced the participant who refused to participate with a subsequent potential participant until the estimated study sample was reached. Eligible participants were physicians and nurses with at least a bachelor's degree in medicine or nursing working in governmental hospitals in Kassala State and having at least 2 years of work experience. All participants were approached, the voluntary and anonymous nature of their participation was explained, and informed consent was obtained. A pen-and-paper self-administered questionnaire was used to collect the data from the participants, emphasizing that the data collection times did not interfere with the process of providing care to patients. Data was collected between August 2019 and February 2021. Of the total study participants, 61% were men and 39% were women. Participant ages ranged from 25 years to 54 years, with a mean age of 36.4 (SD = 7.6) years. The majority (75.2%) were ever married. Almost 60% were physicians, 71% had a bachelor's degree, and 29% had postgraduate degrees. Participants' years of work experience ranged from 1 to 19, with a mean of 7.8 (SD = 5.4) years.

### Measures

The HIV stigma subscale measures used in this study were mainly based on Stein and Lie's HIV stigma scale for healthcare providers (13). Answers were provided on a seven-point Likert scale ranging from 1 (disagree) to 7 (Agree). This scale is frequently used to measure HIV stigma constructs among healthcare providers and is considered valid and reliable (44, 45).

Intention to discriminate against people with HIV was measured using four-item which measures healthcare providers discrimination intent at work (13). All items were reverse-coded, and a high score is indicative of a high intention to discriminate. An example item is “willing to work with HIV-positive patients.” The omega coefficient (ω) was 0.85.

Prejudiced attitudes were measured using a four-item from Stein and Lie's HIV stigma scale, which measures prejudicial attitudes among healthcare providers (13) (ω = 0.77). The items are “People who got HIV through sex and drug use got what they deserved.” “Infected through commercial sex deserve sympathy” (reverse-coded), “Infected through drug use deserve sympathy” (reverse-coded), and “People who behave promiscuously should be blamed for HIV.” A higher score indicates strong prejudicial attitudes.

Internalized shame about HIV was measured using three-item from Stein and Lie's HIV stigma scale, which measure internalized shame about HIV among healthcare providers (ω = 0.76) (13). A high score is indicative of a high internalized shame about HIV. An example item is “I feel ashamed if a relative got HIV.”

Fear of HIV was measured using three items developed by Stein and Lie, which measures healthcare providers' fear of HIV (ω = 0.76) (13). A high score is indicative of a high fear of HIV. An example item is “I afraid of people with HIV.”

Belief that patients with HIV deserve good care was measured using three-item from Stein and Lie's HIV stigma scale, which measures healthcare beliefs provision of good care for patients who got HIV through blood donation, commercial sex, and drug use (ω = 0.84) (13). All three items were reverse coded, and a high score indicates a higher belief that people with HIV do not deserve good care. An example item is “people who got HIV through sex deserve good care.”

Sociodemographic variables included age, gender, and marital status. Occupational characteristics included profession (physician or nurse), academic qualification (bachelor, master, and doctorate), and years of work experience.

### Analysis

The study participants' characteristics were described using frequency, mean, range, and standard deviations. The mean of responses to questions on each subscale was used to compute the composite subscale scores for intention to discriminate against people with HIV, as well as stigma-related constructs: prejudiced attitudes, internalized shame about HIV, fear of HIV, and belief that people with HIV deserve good care. Pearson correlation coefficients were computed to assess the relationships between the intention to discriminate against people with HIV, prejudiced attitudes, internalized shame about HIV, fear of HIV, and belief that people with HIV deserve good care.

A multiple regression analysis was used to measure the association of the dependent variable, the intention to discriminate against people with HIV, with explanatory variables stigma-related constructs, sociodemographic (age, gender, and marital status), and occupation characteristics (profession, qualification, and year of work experience). For all analyses, confidence intervals and *P*-values were computed. The *P* < 0.05 were considered statistically significant. Variance inflation factor and tolerance were computed to detect multicollinearity levels between the variables used in the multiple regression. The variance inflation factor was below 10, and tolerance was above 0.1, indicating no reason for concerns about multicollinearity.

Statistical Package for the Social Sciences (SPSS) version 23 was used for the analysis of the study data.

## Results

### Descriptive statistics

Table 1 shows the descriptive analysis of demographic and occupational variables of the study participants.

**Table 1:** Sociodemographic and occupational characteristics of study participants (*N* = 387).

**Characteristics**	***n***	**%**	**M**	**SD**	**Range**
Age			36.4	7.61	(25–54)
Gender					
Male	236	61.0%			
Female	151	39.0%			
Marital status					
Ever married	291	75.2%			
Never married	96	24.8%			
Profession					
Physicians	223	57.6%			
Nurse	164	42.4%			
Academic qualification					
Bachelor	276	71.3%			
Postgraduate degrees	111	19.9%			
Years of work experience			7.8	5.41	(1–19)

### Stigma-related constructs descriptive statistics and correlations

Table 2 shows descriptive statistics and correlations between HIV stigma-related constructs. The mean score for “discrimination intent against people with HIV” was 5.19 (SD = 1.34) and the mean score for the belief that patients with HIV do not deserve good care was 4.90 (SD = 1.35). The relationships between HIV stigma-related constructs and intentions to discriminate against people with HIV were positive and statistically significant. Fear of HIV and prejudiced attitudes were positively correlated (*r* = 0.54, *p* < 0.01). The intention to discriminate against people with HIV and the belief that patients with HIV do not deserve good care were positively correlated (*r* = 0.52, *p* < 0.01). The variables internalized shame about HIV and fear of HIV were positively correlated (*r* = 0.31, *p* < 0.01). The mean score of intention to discriminate was 5.19 (SD = 1.34), which indicates that most of the participants hold intentions to discriminate against people with HIV. Regarding the stigma-related construct, the mean score of fear of HIV was 4.65 (DS = 1.39) and the mean of prejudiced attitudes was 4.70 (SD = 1.29) suggesting that more than half of the participants express fear and prejudiced attitudes toward people with HIV.

**Table 2:** Descriptive statistics and correlations for study variables.

	**Variables**	**Mean**	**SD**	**1**	**2**	**3**	**4**	**5**	**Internal consistency**
1.	Prejudiced attitudes	4.70	1.29						0.77
2.	Internalized shame about HIV	4.67	1.27	0.28^**^ [0.18, 0.34]					0.76
3.	Fear of HIV	4.65	1.39	0.54^**^ [0.45, 0.62]	0.31^**^ [0.21, 0.39]				0.76
4.	Patients with HIV do not deserve good care ^**R**^	4.90	1.35	0.47^**^ [0.38, 0.54]	0.35^**^ [0.25, 0.43]	0.43^**^ [0.34, 0.51]			0.84
5.	Discrimination intent against people with HIV ^**R**^	5.19	1.34	0.48^**^ [0.39, 0.56]	0.37^**^ [0.27, 0.45]	0.53^**^ [0.44, 0.61]	0.52^**^ [0.43, 0.59]		0.85

M and SD are used to represent mean and standard deviation. ^R^Refers to reverse scores. Values in square brackets indicate the 95% confidence interval for each correlation.

* *p* < 0.05.

** *p* < 0.01.

Table 3 shows the results of the regression analysis. The results revealed a statistically significant model [F (10/376) = 36.44, *P* < 0.001], with a *R*^2^ of 0.49. This indicates that stigma-related constructs, sociodemographic, and occupation variables accounted for 49% of the variance in the intention to discriminate against people with HIV among the study participants. The regression coefficient for gender was 0.29, with a *P* < 0.05, indicating that females score high on their intention to discriminate against people with HIV than males. The regression coefficient for the profession (physician vs. nurse) variable was −0.387, *p* < 0.001, suggesting that physicians scored lower than nurses in terms of intention to discriminate against people with HIV. The result also indicated that healthcare providers with postgraduate degrees are more likely to have the intention to discriminate against people with HIV compared to those with bachelor's degrees. Healthcare providers' age and marital status variables were not statistically significant associated with the intention to discriminate against people with HIV.

**Table 3:** Multiple regression analysis: sociodemographic, occupational characteristics, and stigma-related constructs associated with intention to discriminate against people with HIV.

**Variables**	**B**	**SE**	**t**	**95% CI**		**P**
				**LL**	**UL**	
**Sociodemographic and occupational characteristics**						
Age	0.013	0.008	1.63	−0.003	0.028	0.103
Gender (ref: female)	0.290	0.127	2.28	0.041	0.539	<0.05
Married status (ref: ever)	0.045	0.133	0.342	−0.216	0.308	0.446
Profession (ref: nurse)	−0.387	0.104	−3.706	−0.592	−0.182	<0.001
Qualification (ref: postgraduate degrees)	−0.337	0.117	−2.881	−0.564	−0.107	<0.001
Years of work experience	−0.052	0.011	−4.747	−0.074	−0.031	<0.001
**Stigma-related constructs**						
Prejudiced attitudes	0.129	0.050	2.596	0.031	0.226	<0.05
Internalized shame about HIV	0.115	0.043	2.679	0.032	0.201	<0.01
Fear of HIV	0.252	0.045	5.580	0.163	0.341	<0.001
Patients with HIV do not deserve good care	0.221	0.047	4.901	0.132	0.310	<0.001

F (10/376) = 36.441, *P* < 0.001. *R*^2^ = 0.49, CI, confidence interval; LL, lower limit; UL, upper limit. B, Unstandardized Coefficients. SE, Standard Error of Unstandardized Coefficients. P, *p*-value.

In the multiple regression analysis for the stigma-related constructs and intention to discriminate against people with HIV, prejudiced attitudes, internalized shame about HIV, fear of HIV, and belief that patients with HIV do not deserve good care were statistically and significantly associated with the intention to discriminate against people with HIV. The regression coefficient for fear of HIV was found to be 0.25, with a *P*-value of < 0.001, indicating a positive relationship between fear of HIV and the intention to discriminate against people with HIV. Additionally, the regression coefficient for the belief that patients with HIV do not deserve good care was 0.22, *P* < 0.001, indicating a that the belief that patients with HIV do not deserve good care is positively associated with healthcare providers' intentions to discriminate against people with HIV.

## Discussion

This study suggests that healthcare providers' intention to discriminate against people with HIV is associated with HIV stigma-related constructs and their sociodemographic characteristics. Specifically, healthcare providers who hold prejudicial attitudes toward people with HIV, greater internalized shame about HIV, high fear of HIV, and belief that people with HIV do not deserve good care they express greater intention to discriminate against people with HIV. Health care providers' gender, profession, qualification, and years of work experience are also associated with the intention to discriminate against people with HIV. This study provides insight for understanding healthcare providers' intention to discriminate and highlights the need to decrease the intention to discriminate against people with HIV among healthcare providers.

Our results found that the intention to discriminate (M = 5.7, SD = 1.34) against people with HIV is high among the study participants. This finding is higher than results of a similar study conducted among physicians in Malaysia (M = 1.6, SD = 0.64) (44). The high prevalence of HIV in Malaysia (53) compared to Sudan (8) could explain this difference, as it provides healthcare providers with more opportunities to interact with HIV-positive individuals compared to those in Sudan. More frequent contact with people with HIV would result in less stigma and discrimination against them (54).

The study's findings indicated that healthcare providers' prejudiced attitudes toward people with HIV are associated with an intention to discriminate against them. However, in the presence of prejudiced attitudes and intention to discriminate, the service offered to people with HIV is compromised and may prevent them from receiving care (36). Prejudiced attitudes toward people with HIV may come from negative cultural beliefs and thoughts (55, 56), yet such unfavorable beliefs about people with HIV are common among Sudanese communities (14). Promoting HIV-related knowledge and interaction with people with HIV can produce more favorable attitudes toward people with HIV among healthcare providers (57) and in turn lower discrimination intent against people with HIV. Our finding is in agreement with results of a study from China among healthcare providers, which found that prejudicial attitudes significantly predict their discriminatory practices at work (58).

Our study has also shown that healthcare providers with greater fear of HIV express more discrimination intent against people with HIV. Health care providers' fear of contracting HIV infection can be due to poor knowledge about universal infection precautions (59) and universal precaution guidelines (60, 61). Promoting universal precaution education and practices may help to decrease healthcare providers' HIV-related fears (62). The concerns of occupational risk of HIV among healthcare providers can also be reduced by making infection control resources available and accessible. According to a previous study, fear of HIV among health care providers is related to the intention to discriminate against people with HIV (26).

The study results also indicated that healthcare providers who strongly disagree that people with HIV deserve good care express a greater intent to discriminate against them. This finding is in line with a previous study among medical students in Malaysia, which suggested an association between intention to discriminate and the belief that people with HIV do not deserve good care (45). The belief that people with HIV do not deserve good treatment may be based on negative moral judgment about them. This is consistent with a previous study in another context, which shows that nurses' moral judgment about their clients determines whether or not to provide service (63). The moral judgment can be influenced by immoral behaviors that violate social norms, such as commercial sex and drug use, and the diseases associated with these behaviors (64).

This study found that physicians were less likely than nurses to discriminate against people with HIV. Nurses' higher intention to discriminate against people with HIV could be attributed to their insufficient awareness of HIV compared to physicians. A study of Sudanese healthcare providers reveals that nurses have less HIV-related knowledge than doctors (7). A previous study also found that nurses have limited knowledge of how to interact with people with HIV (65). Nurses' limited knowledge of HIV and interactions with people with HIV may have a negative impact on the quality of care they give (66). Our findings are consistent with a study conducted in Egypt on stigma and prejudice against people with HIV, which revealed that nurses discriminate against people with HIV more than doctors (47).

Furthermore, the study's findings indicate that male health care providers were less likely than females to have intentions to discriminate against people with HIV. This finding is consistent with previous studies on stigma among healthcare providers, which indicated that male healthcare providers are less likely to discriminate against people with HIV than females (67, 68). Partly, this result may be related to personal values and views held by female healthcare providers that patients with HIV are infected because of their behavior, which may reflect in discrimination against people with HIV (69).

Another finding was that healthcare providers with more years of working experience were less likely to have the intention to discriminate against people with HIV than those with fewer years of work experience. Health care providers with more years of work experience are more likely to be exposed to HIV-related information, patients, and training, consequently leading to less discrimination of people with HIV. Previous studies suggested an inverse relationship between years of working experience and discrimination (15, 70).

In addition, our study results revealed that healthcare providers with postgraduate degrees showed higher intention to discriminate against people with HIV than those with bachelor's degrees. Similar results were found in a study conducted among healthcare workers in India, in which increases in medical education were found to be associated with high stigmatization and discrimination against people with HIV (71).

Although our study provides insights on predictors of intention to discriminate against people with HIV among healthcare providers, there are some limitations that need to be considered in interpreting its findings. The study only included physicians and nurses from one geographical area, so it is important to take caution when generalizing the study findings to other regions and healthcare providers. The study used a self-reported questionnaire, and social desirability bias may occur. Much effort was made to overcome social desirability bias, including assuring participants about the confidentiality and anonymity of their responses and completing the questionnaire in private settings. We expected this could reduce their hesitancy to report their intention to discriminate against people with HIV.

## Conclusions

This study showed healthcare providers hold intention to discriminate against people with HIV. The healthcare providers' intention to discriminate against people with HIV was associated with prejudiced attitudes, fear of HIV, internalized shame about HIV, and the belief that people with HIV do not deserve good care. Also, female healthcare providers, nurses, and those with postgraduate degrees and more years of work experience were more likely to have intentions to discriminate against people with HIV. This addresses the need to reduce healthcare providers' intentions to discriminate against people with HIV by a holistic approach that targets HIV stigma-related constructs, considering healthcare providers' sociodemographic and occupational characteristics.

## Data Availability

The raw data supporting the conclusions of this article will be made available by the authors, without undue reservation.
